# Physiological Responses of the Firefly *Pyrocoelia analis* (Coleoptera: Lampyridae) to an Environmental Residue From Chemical Pesticide Imidacloprid

**DOI:** 10.3389/fphys.2022.879216

**Published:** 2022-06-15

**Authors:** Yi-zhe Wang, Cheng-quan Cao, Dun Wang

**Affiliations:** ^1^ State Key Laboratory of Crop Stress Biology for Arid Areas, Northwest A&F University, Yangling, China; ^2^ College of Life Science, Leshan Normal University, Leshan, China

**Keywords:** Pyrocoelia analis, imidacloprid, toxicology, antioxidant enzyme activity, tissue structure

## Abstract

Imidacloprid, a neonicotinoid insecticide, is widely applied to control insect pests across a broad spectrum. Though the impact of residues from this chemical pesticide on non-target organisms in the field has been reported, it was not well characterized across a wide range of ecosystems, especially for some species considered as environmental indicators that live in forests. The effects of sublethal dose of imidacloprid on firefly, *Pyrocoelia analis*, were analyzed physiologically and biochemically in this study to better understand the impact of chemical pesticide application on environmental indicators such as fireflies. After imidacloprid treatment, the midgut tissues of the larva presented an abnormal morphology featured as atrophy of fat body cells, shrinking cells, and the destruction of a midgut structure. The activities of antioxidant enzymes, superoxide dismutase, catalase, and peroxidase were noticeably increased during early exposure to sublethal imidacloprid and then decreased at later stages. The malondialdehyde content significantly increased after 12 h of exposure to imidacloprid compared with the control. Similarly, the enzyme activities of polyphenol oxidase and acetylcholinesterase were increased after the imidacloprid treatment and then decreased at the later stage. In summary, a sublethal dose of imidacloprid caused destructive change in the tissue structure, and this damage was followed by an excessive reactive oxygen species that could not be eliminated by antioxidant enzymes. Our results indicated that the residues of imidacloprid might cause severe toxicity to non-target insects in the environment even far away from the agro-ecosystem where the chemicals were applied.

## Introduction

Since imidacloprid [1-(6-chloro-3-pyridylmethyl)-N-nitroimidazolidin-2-ylideneamine] was launched in 1991 by Bayer, it has become one of the most widely used neonicotinoid insecticides with a market share of more than 25% of global pesticide sales due to its high efficiency and broad spectrum (Bass et al., 2015; Kohl et al., 2019). Since its introduction, environmental problems caused by the widespread use of imidacloprid have become increasingly prominent (Morrissey et al., 2015; Pietrzak et al., 2020; Thompson et al., 2020). Several studies have documented that these neonicotinoids persist in the environment for a long time and the contents of neonicotinoid pesticides in different farmlands were all present at the ng/g level (Li et al., 2018; Thompson et al., 2020). Imidacloprid can competitively bind to nicotinic acetylcholine receptors (nAChRs), and the postsynaptic nAChRs are blocked irreversibly, causing the continuous conduction of nerve impulses and thus producing lethal effects (Matsuda et al., 2001; Cartereau et al., 2021). Apart from acting on target organisms, imidacloprid can be dispersed in other non-target organisms and accumulate in the environment by different types of applications (Cloyd and Bethke, 2011; Goulson, 2013; Sharma et al., 2019; Singh and Leppanen, 2020). Most of the residuals are nonbiodegradable and toxic, and several studies have documented that imidacloprid can persist in the environment for a long time (Brunet et al., 2004; Flores-Cespedes et al., 2012) and the contents of neonicotinoid pesticides in different farmlands are present at the ng g^−1^ level (Li et al., 2018; Thompson et al., 2020).

In previous studies, the impact of imidacloprid on non-target species has been investigated, and we know that imidacloprid leads to changes in physiological and biochemical parameters (Vohra et al., 2014; Siregar et al., 2021), Seifert and Stollberg (2005) performed interactions of imidacloprid with the nAChRs of embryonic frog muscle cells and found that imidacloprid induces the contraction of embryonic frog muscle at doses as low as 3.3 × 10^–7^ M. Xia et al. (2016) found that the cysts of the loach tests were disorganized and the interstitial tissue was increased when the loach was exposed to imidacloprid. Moreover, the low dose of imidacloprid resulted in marked irregularities and fragmentation of midgut cells in earthworms (Dittbrenner et al., 2011). The exposure to imidacloprid led to changes in the biochemical parameters of the Pacific white shrimp *Litopenaeus vannamei*, which caused oxidative stress, retarded growth, and immune and tissue damage (Fu et al., 2022). Pervez and Manzoor (2020) found that physiological and behavioral functions for normal foraging and colony maintenance were modified in honeybees (*Apis mellifera*) exposed to 1.25, 2.5, and 5 mg L^−1^ imidacloprid, respectively.

Exposures to low concentrations of imidacloprid have been reported to change the physiological responses of organisms (Zhang et al., 2014). Moreover, the AChE activity, antioxidant enzyme activities, and MDA contents were considered as the significant biomarkers to investigate the influence of pollutants (Deng et al., 2021; Guo et al., 2022). Under the stress of imidacloprid, the acetylcholinesterase in the synapse cannot metabolize imidacloprid in the postsynaptic nAChRs, and this caused continuous nerve impulses and induced oxidative stress (Jepson et al., 2006; Janner et al., 2021). Reactive oxygen species (ROS) can be produced in living organisms, but excessive ROS can also result in oxidative stress and lipid peroxidation. Malondialdehyde (MDA) is the end product of lipid peroxidation caused by ROS, and the MDA level may also indicate the level of ROS (Chen J. et al., 2015). To protect cells from oxidative stress, some antioxidant and detoxifying enzymes scavenge the overproduced ROS, such as superoxide dismutase (SOD), catalase (CAT), peroxidase (POD), and polyphenol oxidase (PPO) (Maity et al., 2008; Chen et al., 2018). Thus, the toxic effects of the pesticide on organisms can be indicated by the level of MDA and enzymatic activities.

The firefly is an important species and is considered to be an environmental indicator, has ornamental value, and is also useful to control pests, such as slugs and snails (Ohba, 2005; Fang et al., 2013; Fu and Benno Meyer-Rochow, 2013; Osozawa et al., 2015; Guo, 2017; Sato, 2019). *Pyrocoelia analis* belongs to the order Coleoptera, family Lampyridae (Fu, 2014) and is a common local firefly in most mountain regions of west China. Fireflies are very sensitive to pollution and insecticides (Pearsons et al., 2021) and fireflies have been used as ecological indicators due to their diverse luminescence and flashing behaviors (Fu et al., 2017; Zhang et al., 2019; Zhang et al., 2021). Over the past decades, wild firefly populations have declined globally, and some species were even threatened with extinction (Fu et al., 2017; Chatragadda, 2020; Lewis et al., 2020; Mbugua et al., 2020; Pearsons et al., 2021), and pesticide use was considered as one of the most serious threats to fireflies (Lewis et al., 2020). However, fewer studies have investigated the influence of chemical pesticides on fireflies at very low doses in the environment. In this study, we examined the toxicity of imidacloprid to fireflies, the detoxifying enzyme activity, antioxidant activity, and histological sections were analyzed to understand the effects of sub-lethal dose of imidacloprid on *Pyrocoelia analis*. This study reported the physiological response of imidacloprid to *Pyrocoelia analis*, and we provide a fundamental understanding for the physiological response of fireflies to chemical pesticides.

## Materials and Methods

### Chemicals

Imidacloprid (≥95.5% purity) was purchased from Shandong Sino-agri United Biotechnology Co., Ltd, China. The other chemicals used in the experiment were of analytical grade and were purchased from Tiancheng Chemical Company in Yangling (Shannxi, China).

### Insects

All the larvae of one firefly species, *Pyrocoelia analis*, were from the Firefly Breeding Base in Hainan, China. The larvae of the firefly were domesticated for 2 weeks in an insect rearing room (26 ± 1°C, 70% relative humidity and a 12:12 light/dark cycle) in a laboratory and were fed with a snail species that was collected at the Northwest A&F University. After acclimation, some of the third instars larvae were selected and moved to transparent plastic boxes (30 cm × 20 cm × 10 cm) after comparing them with larval developmental parameters available from previous growth experiments. Every transparent plastic box had 60 larvae and the bottom of the box was covered by a moist filter paper.

Determination of Sublethal Concentration

We dissolve imidacloprid with acetone, and diluted it with water at different concentrations (0.025, 0.05, 0.1, 0.2, and 0.4 mg/L). To determine the sublethal concentration of imidacloprid in larvae of *P. analis*, different imidacloprid concentrations were sprayed on the third instar larvae. Three replicates of 30 larvae from each replicate were used for each treatment and a solvent group without imidacloprid was used as a control. The mortality was recorded after 0, 3, 6, 12, 24, 48, and 72 h. The calculation of the toxicity regression equation and LC_10_ was based on corrected mortality.

### Experimental Design

According to the result from the determination of sublethal concentrations, the concentration of 1 mg/L was set in the experiment. Larvae were placed in each transparent plastic box with a filter paper, and the imidacloprid of 1 mg/L concentration was sprayed on the larvae until the filter paper was moist (to make the humidity in the box at 75%). The pesticide treatment group was all tested with triple replicates of 100 larvae in each replicate. All the experiments were maintained under laboratory conditions at 26 ± 1°C under a 12:12 light/dark cycle for 3 days. After the application of imidacloprid, the larvae were collected at the 0, 3, 6, 12, 24, 48, and 72 h for observation of poisoning symptoms, histopathological studies, analysis in enzyme activities (SOD, POD, PPO, CAT and AChE activities), and MDA assays.

### Recording of Poisoning Symptoms

The larvae of *P. analis* collected at 0h, 3h, 6h, 12h, 24h, 48h, and 72 h were observed for post-pesticide treatment for the recording of poisoning symptoms. The recording of symptoms was conducted immediately after collection. During this observation, we recorded the curling degree of the larvae, the retraction position of the head, and the illuminator characteristics. The larvae were then photographed in a bright field and dark field, respectively.

### Histological Examination

The larvae were collected in a 10 ml tube at different times compared to the control group and treatment group, and placed in a 4% paraformaldehyde solution over 24 h. Then, the samples were dehydrated in ascending grades of alcohol, cleared in xylene, and embedded in paraffin wax (75% alcohol 1H, 85% alcohol 1h, 95% alcohol 1h, 95% alcohol 1H, 95% alcohol 1H, anhydrous ethanol I 1h, anhydrous ethanol II 1H, xylene 1h, xylene 1H, wax I 1 h, and wax II 1H). The samples soaked in wax were embedded in the embedding machine. After cooling in a −20° freezer, the wax blocks were removed from the embedding frame after solidification and trimmed. Approximately 3 μm thin sections were cut on a rotatory microtome (RM 2016, Leica Germany). The ribbons with tissues were stretched and fixed to a clean albumenized glass slide and warmed to 60°C. These glass slides were, then, placed in an incubator overnight for stretching and the removal of bubbles. The slices were sequentially placed in xylene I for 8 min, xylene II for 8 min, anhydrous ethanol I for 6 min, anhydrous ethanol II for 6 min, 95% alcohol for 6 min, 85% alcohol for 6 min, 75% alcohol for 5 min, and rinsed with running water for 5 min. Then, the slices were stained with Harris hematoxylin for 3–8 min and rinsed with running water to remove the excess fuel. After that, the samples were differentiated with 1% hydrochloric acid alcohol for a few seconds and rinsed with tap water. The sections were then stained in eosin staining solution for 1–3 min, they were dehydrated in ascending grades of alcohol (75% alcohol 30s, 85% alcohol 30s, 95% alcohol I 1min, 95% alcohol II 2 min, anhydrous ethanol I 5 min, and anhydrous ethanol II 5 min), cleared in xylene (xylene I 5 min and xylene II 7 min), and covered with neutral gum. The slides were photographed and examined under a microscope (Ds-fi3, High-definition Color Microscope Camera—Nikon Japan).

### Enzyme Activity and MDA Assays

The larvae of *P. analis* were collected at 0, 3, 6, 12, 24, 48, and 72 h post-pesticide treatment and then were frozen in the −80°C freezer. Three biological replicates were frozen at each time point. Once the sample collection was completed, each replicate was weighed and then put into 2 ml tubes with a steel bead and were grinded to a powder state using the Mixer Mill MM 400 (Retsh GmbH, Germany). Then, we added 0.05 M phosphate-buffered saline (PBS) to the tube and mixed it well. The homogenate was centrifuged at 2000 r/min at 4°C for 10 min and the supernatant was used for the measurement of the MDA content, enzyme activities, and protein concentrations. The protein concentration was measured using the method of Bradford (1976). All experimental steps were carried out in accordance with the kit instructions. All the kits used for the enzyme activity tests and MDA contents were purchased from the Jiancheng Bioengineering Institute (Nanjing, China). The results of these enzymatic assays were given in units of enzymatic activity per milligram of protein (U/mg prot), and the MDA content was defined as a nanomole TBA reactive substance per milligram protein (nmol/mg prot). The enzyme activity was the average of the three biological replicates.

### Statistical Analysis

All statistics were conducted by SPSS software (SPSS 26.0). Parametric tests were preceded by tests to evaluate the homogeneity of variances. Differences between the treatment and controls regarding enzyme activity and MDA content were determined using the independent-samples *t*-test. A one-way analysis of variance (ANOVA) followed by a LSD multiple comparison test was used to analyze the enzyme activity and MDA content of each group at different times after exposures to imidacloprid, and the significant differences between the groups were compared. Data are expressed as the mean ± standard error (SE). *p* < 0.05 was regarded as statistically significant, and *p* < 0.01 was considered as statistically very significant.

## Results

### Toxicity of Imidacloprid Against *P. analis*

Under laboratory conditions, imidacloprid is highly toxic to third-instar larvae of *P. analis.* The toxicity regression equation (72 h) was y = -2.123 + 7.892x with a fiducial limit of 95%, and the chi-squared test of goodness of fit for this equation was 1.302. The imidacloprid sublethal concentration LC_10_ was calculated as 0.1 mg/L through the toxicity regression equation, and the fiducial limit of 95% was 0.056—0.144 mg/L.

### Poisoning Symptoms and Luminant Characters

**FIGURE 1:**
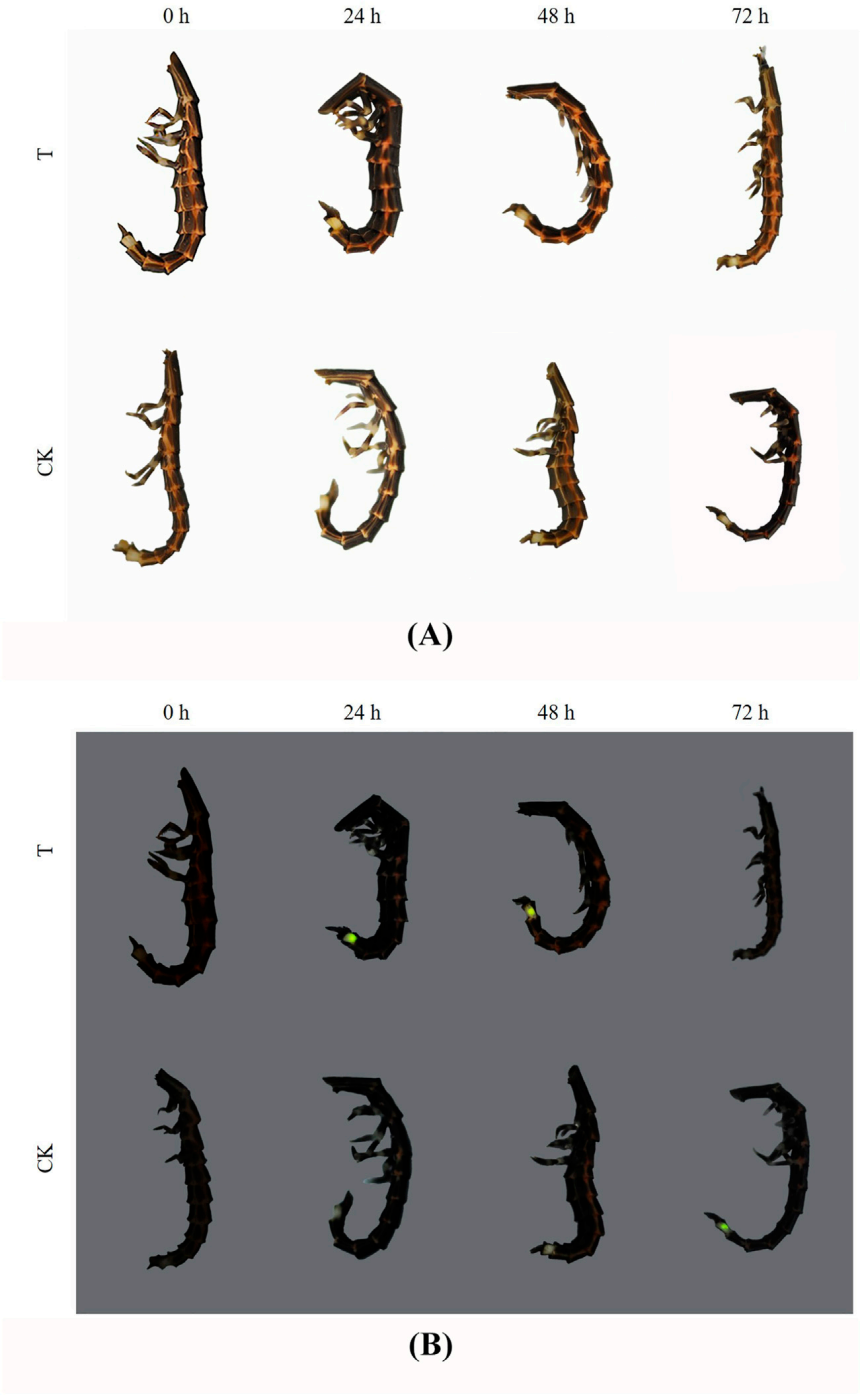
Poisoning symptoms and luminant characters of *P. analis* at different times. **(A)** Photo in the bright field **(B)**. Photo in the darkfield. T: the treated group, CK: the control group.

The larvae of *P. analis* showed hyperactivity and began moving quickly at the beginning of exposure to imidacloprid. The poisoning symptoms were observed in larvae of *P. analis* when exposed to imidacloprid after 6 h (Figure 1). The heads of the larvae were bent toward the inside of the thorax and the bodies were twisted into a 3D-shape, accompanied with intermittently trembling. After 24 h post-treatment, the larvae laid on their side and the heads of these insects were almost at right angles to the prothorax. Their legs were constantly shaking, and the illuminators began to emit luminescence continuously after responding to gentle stimulation. However, the control group did not respond to gentle stimulation. This persistent glow phenomenon was observed from 12 to 48 h after treatment and the luminous brightness gradually decreased with the increased exposure time to imidacloprid. After 72 h, the head of the larva protruded from the prothorax, and the antennae and legs convulsed continually, then the insects regurgitated gastric juice and the illuminator no longer glowed.

### Tissue Histological Profiles

**FIGURE 2:**
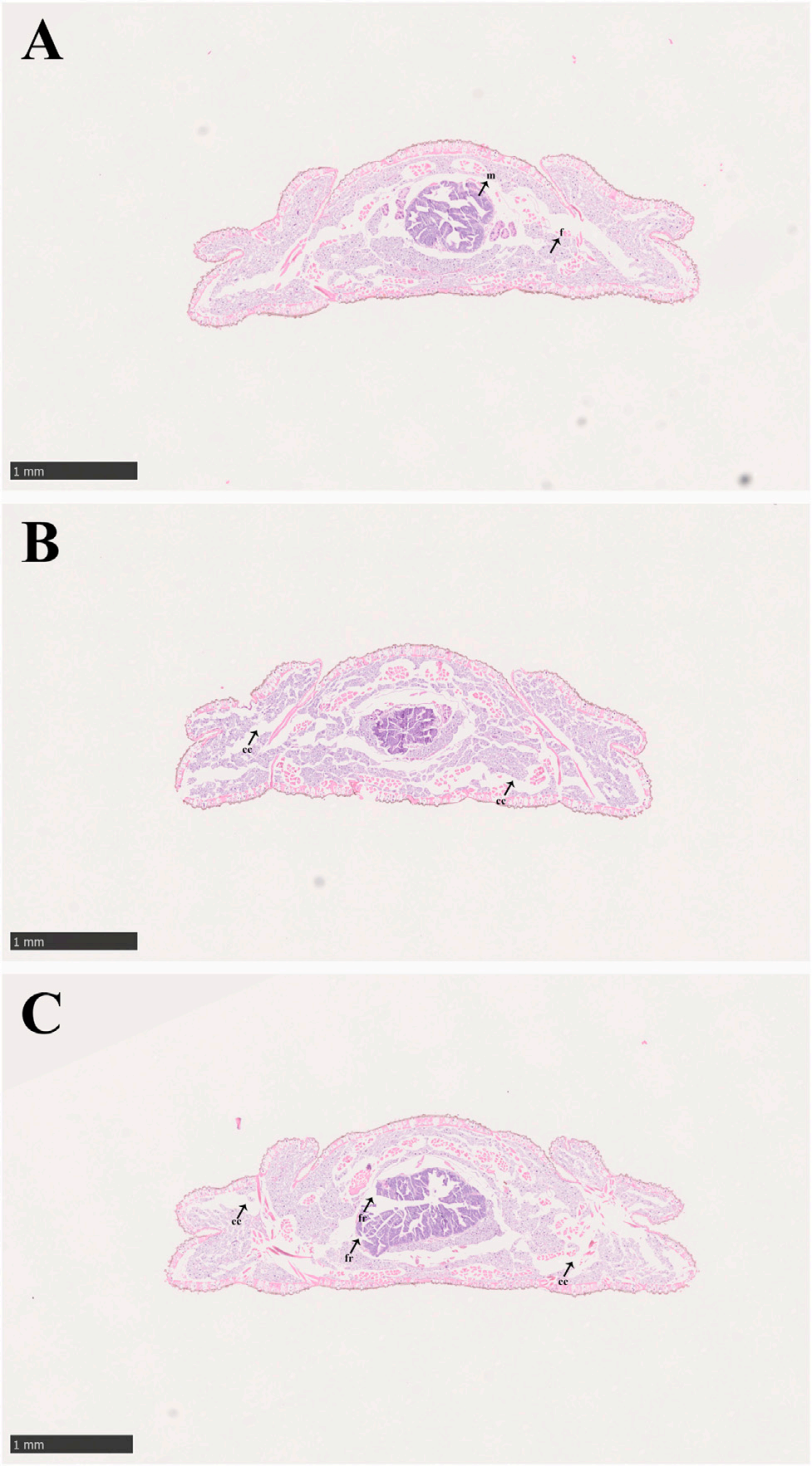
Midgut structure of *P. analis.* **(A)** Control group, **(B)** after 48 h of imidacloprid exposure, **(C)** after 72 h of imidacloprid exposure. m, midgut cell; f, normal fat body; cc, cellular compartmentation; fr, fragmentation of the midgut.

The condition of the midgut tissue deteriorated with increasing imidacloprid exposure time (Figure 2). In the control group, the tissues of midgut in larvae of *P. analis* were normal and the structure of the midgut was complete. The cells of the tissues were clear, and the adipocytes around the midgut were normal and tightly arranged (Figure 2A). However, the larger gap between the cells was observed in the midgut and adipose tissue during the treatment after 48 h of exposure (Figure 2B). With increasing imidacloprid exposure time, we observed shrinking cells, fragmentation of the midgut, and the increased occurrence of intercellular spaces (Figure 2C).

**FIGURE 3:**
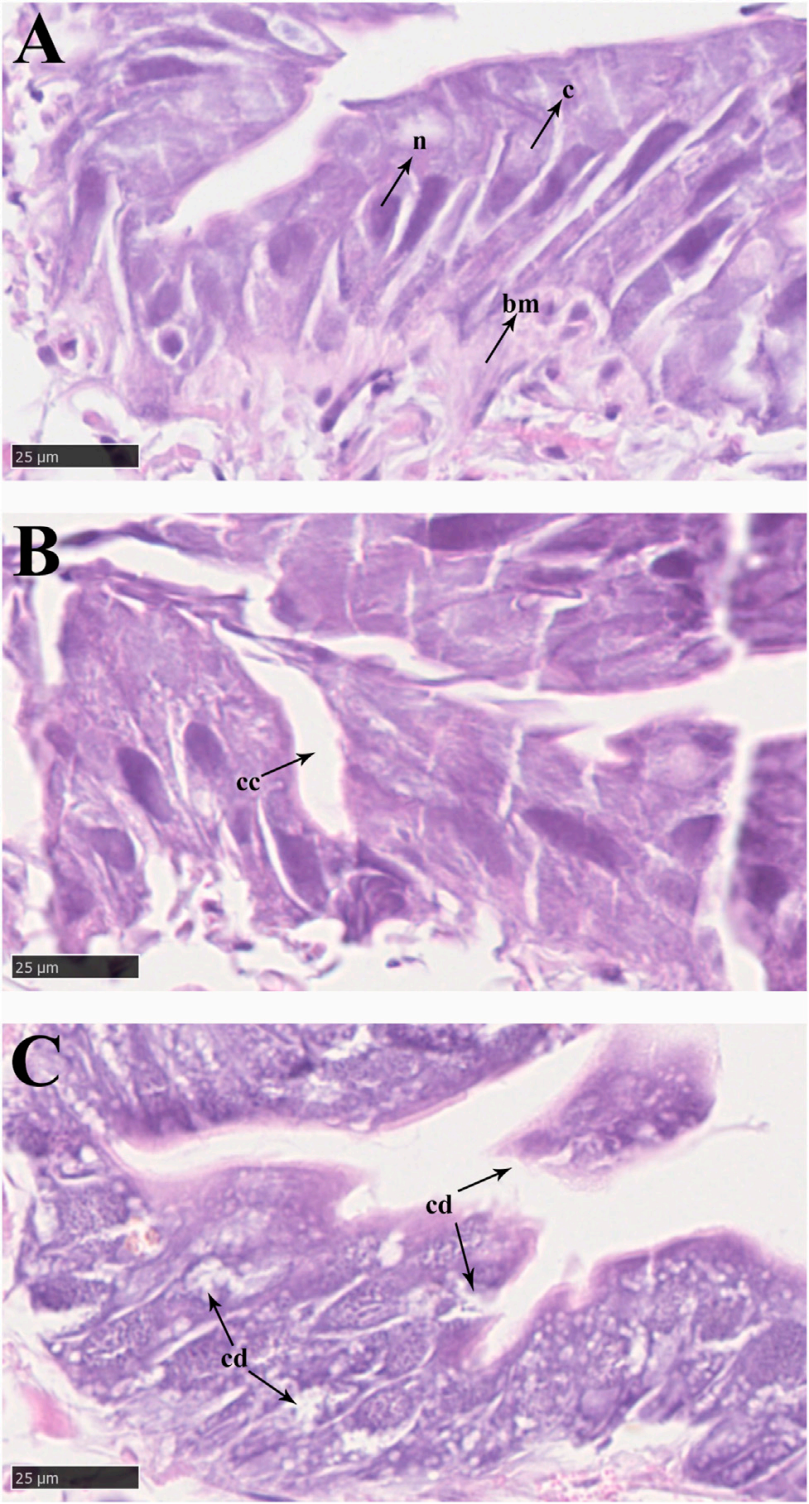
Epithelial cells in the midgut of *P. analis.* **(A)** Control group. **(B)** After 48 h of imidacloprid exposure. **(C)** After 72 h of imidacloprid exposure. bm, basement membrane; c, cytoplasm; n, nucleus; cc, cellular compartmentation; cd, cell debris.

**FIGURE 4:**
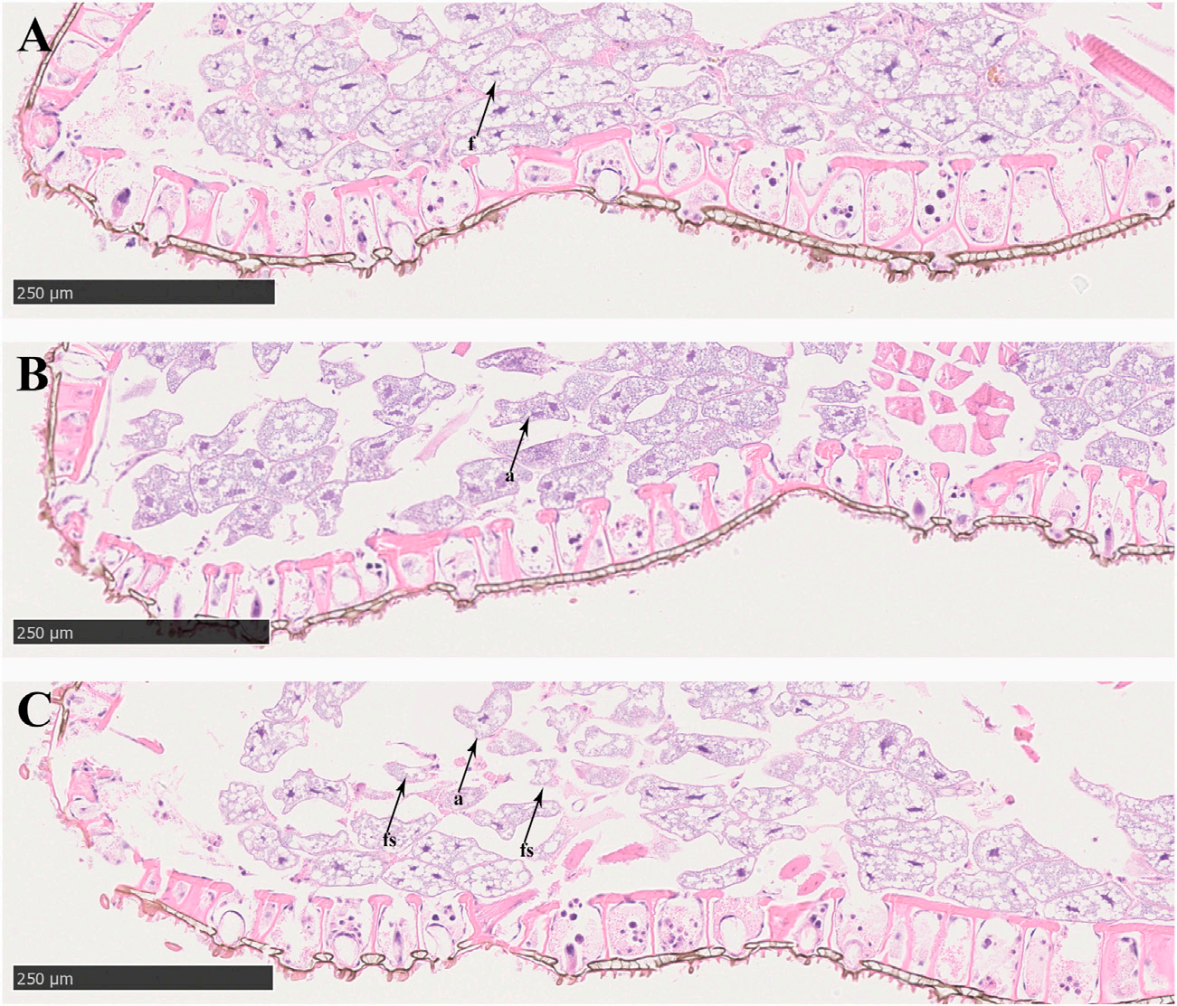
Fat body cell of *P. analis.* **(A)** Control group, **(B)** after 48 h of imidacloprid exposure, **(C)** after 72 h of imidacloprid exposure. f, normal fat body cell; a, atrophy of fat body cell; fs, the fragmented shrinking cell.

In the control groups, the shapes of midgut epithelial cells were neatly arranged and dense, and the nuclei were clearly visible (Figure 3A). With increasing exposure time to imidacloprid, the cell structure was unclear, increased cellular compartmentation and irregularity of nuclei shape also arose in midgut tissue after 48 h (Figure 3B). Furthermore, after 72 h of imidacloprid exposure, the degradation of the cellular compartmentation was observed in midgut tissue, many midgut cells were destroyed, and there was some cell debris in the lumen (Figure 3C). Moreover, the atrophy of fat body cells was also observed after 48 h post-exposure and the shrinking cells fragmented after 72 h of exposure (Figure 4).

### AChE Activity

**FIGURE 5:**
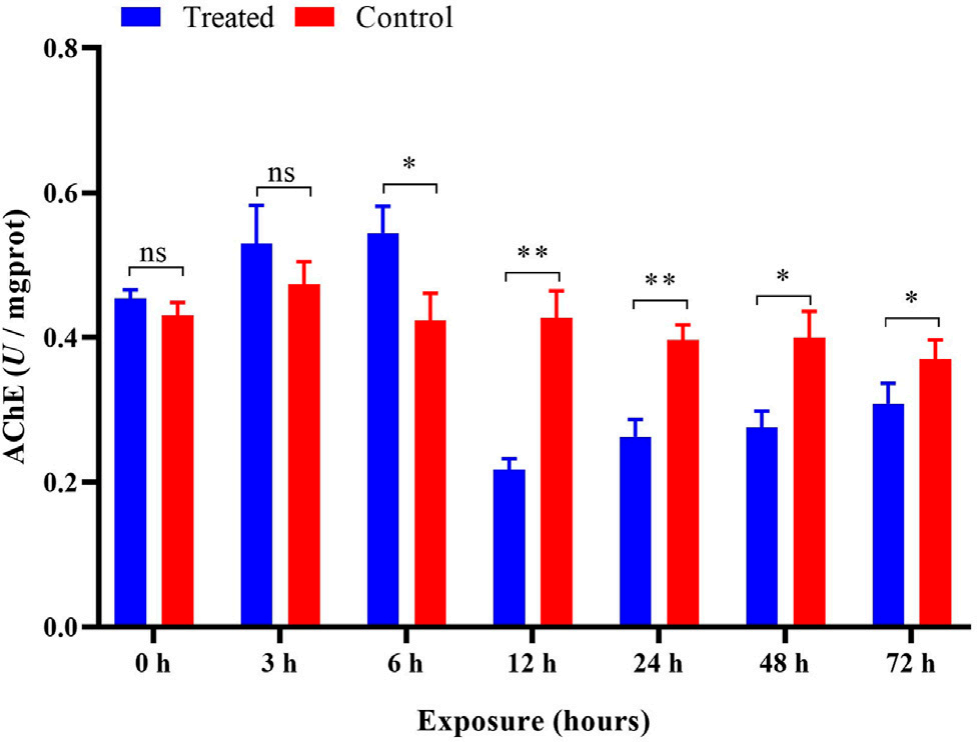
Effects of imidacloprid on the AChE activity of *P. analis*. Each bar represents the mean of three replicates, and the error bars represent the standard deviation (SD). Significant values (*p < 0.05, **p < 0.01) refer to the difference between exposed samples and the controls.

Compared with the control, no significant (*p* > 0.05) changes were observed in AChE activity during the 0 h and after 3 h of pesticide exposure. However, after exposure to imidacloprid at 6 h, the activity of AChE was significantly (*p* < 0.05) higher than that of the control group, reaching approximately 129% of the value in the control, and they were significantly inhibited at 12, 24, 48, and 72 h of exposure (Figure 5). Univariate analysis (ANOVA) revealed significant influences of different duration (*p* < 0.05) on the activity of AChE (Table 1), and AChE activity gradually increased over time and then was significantly inhibited after 12 h in exposed imidacloprid (Figure 5 and Table 1).

**TABLE 1:** Effects of imidacloprid on antioxidant enzymes, detoxifying enzymes, and malondialdehyde content of *P. analis*. larvae.

(h)	SOD (U/mg protein)	CAT (U/mg protein)	POD (U/mg protein)	PPO (U/mg protein)	AChE (U/mg protein)	MDA (nmol/mg protein)
0	20.367 ± 0.568 d	11.900 ± 0.153 e	2.730 ± 0.090 c	5.920 ± 0.407 b	14.133 ± 0.633 c	0.144 ± 0.002 d
3	23.524 ± 0.640 c	12.733 ± 0.067 e	3.219 ± 0.125 b	8.064 ± 0.306 a	16.853 ± 0.480 b	0.144 ± 0.002 d
6	25.087 ± 0.581 c	14.947 ± 0.260 cd	3.697 ± 0.102 a	7.273 ± 0.425 a	18.567 ± 0.691 a	0.159 ± 0.009 c
12	27.527 ± 0.709 b	16.567 ± 0.338 b	2.412 ± 0.173 c	5.211 ± 0.074 b	16.467 ± 0.437 b	0.192 ± 0.004 b
24	31.098 ± 1.849 a	18.567 ± 0.491 a	2.055 ± 0.044 d	3.665 ± 0.236 c	14.347 ± 0.558 c	0.152 ± 0.006 cd
48	19.740 **±** 0.896 d	15.187 ± 0.238 c	2.044 ± 0.072 d	3.955 ± 0.068 c	12.700 ± 0.666 cd	0.227 ± 0.004 a
72	19.984 ± 0.661 d	14.200 ± 0.173 d	1.708 ± 0.072 e	2.406 ± 0.249 d	11.233 ± 0.633 d	0.237 ± 0.001 a

Data are expressed as mean ± SE of three replicates. Different letters (a, b, c, d, and e) indicate statistical differences between groups at the *p* < 0.05 level at the same column.

### Antioxidant Enzyme Activity

**FIGURE 6:**
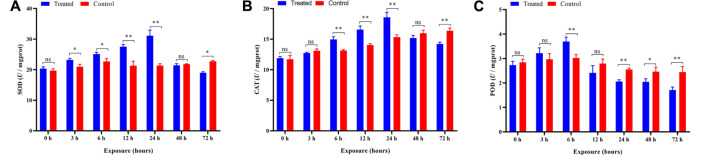
Effects of imidacloprid on the antioxidant activity of *P. analis*. Each bar represents the mean of three replicates, and the error bars represent the standard deviation (SD). Significant values (*p < 0.05, **p < 0.01) refer to the difference between exposed samples and the controls. **(A)** Effects of imidacloprid on the SOD activity of *P. analis*. **(B)** Effects of imidacloprid on the CAT activity of *P. analis*. **(C)** Effects of imidacloprid on the POD activity of *P. analis.*

The activity of SOD was significantly increased (*p* < 0.05 and *p* < 0.01) after 3–24 h and then was inhibited (*p* < 0.05) after 48 h of exposure compared to the control (Figure 6A). The ANOVA revealed significant influences of different duration (*p* < 0.05) on the activity of SOD, and the SOD activity in treatments was increased significantly (*p* < 0.05) with increasing exposure time during 3–24 h (Table 1), indicating that the duration of exposure played a crucial role in affecting the activity of SOD in *P. analis*.

Compared with the control, a significant increase in the CAT activity was observed in the *P. analis* larvae after imidacloprid exposure from 6 to 24 h (*p* < 0.01), while the CAT activity was inhibited significantly (*p* < 0.01) after 72 h of exposure (Figure 6B). The results of the ANOVA showed the significant influences of different duration (*p* < 0.05) on the activity of CAT, the trend of CAT activity increased first and then decreased, while the peak occurred at 24 h after imidacloprid exposure (Table 1).

The activity of the POD was significantly elevated in larvae of *P. analis* exposed to imidacloprid after 6 h of treatment. However, the activity of the POD was significantly lower than that of the control after 24 h, 48, and 72 h (Figure 6C). The results of the ANOVA showed that the significant (*p* < 0.05) variation of POD activity in *P. analis* was observed with the increase of exposure time to imidacloprid, with POD activity increasing at first and then decreasing, and the peak occurred after 6 h (Table 1).

**FIGURE 7:**
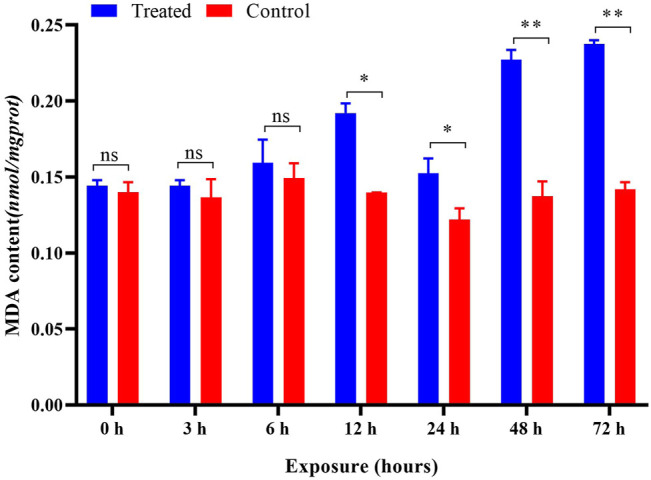
Effects of imidacloprid on the MDA content of *P. analis*. Each bar represents the mean of three replicates, and the error bars represent the standard deviation (SD). Significant values (*p < 0.05, **p < 0.01) refer to the difference between exposed samples and the controls.

After imidacloprid exposure, the content of MDA was significantly higher than that of the control after 6 h of exposure (Figure 7). The results of the ANOVA showed that different duration (*p* < 0.05) played a crucial role in affecting the content of MDA induced by imidacloprid (Table 1). With increasing exposure time, the content of MDA was increased significantly in the treatments from 6 to 12 h, decreased from 12 to 24 h, and then increased from 24 to 48 h. There was no significant difference after imidacloprid exposure between 48 and 72 h (*p* < 0.05) (Table 1).

### Detoxifying Enzyme Activity

**FIGURE 8:**
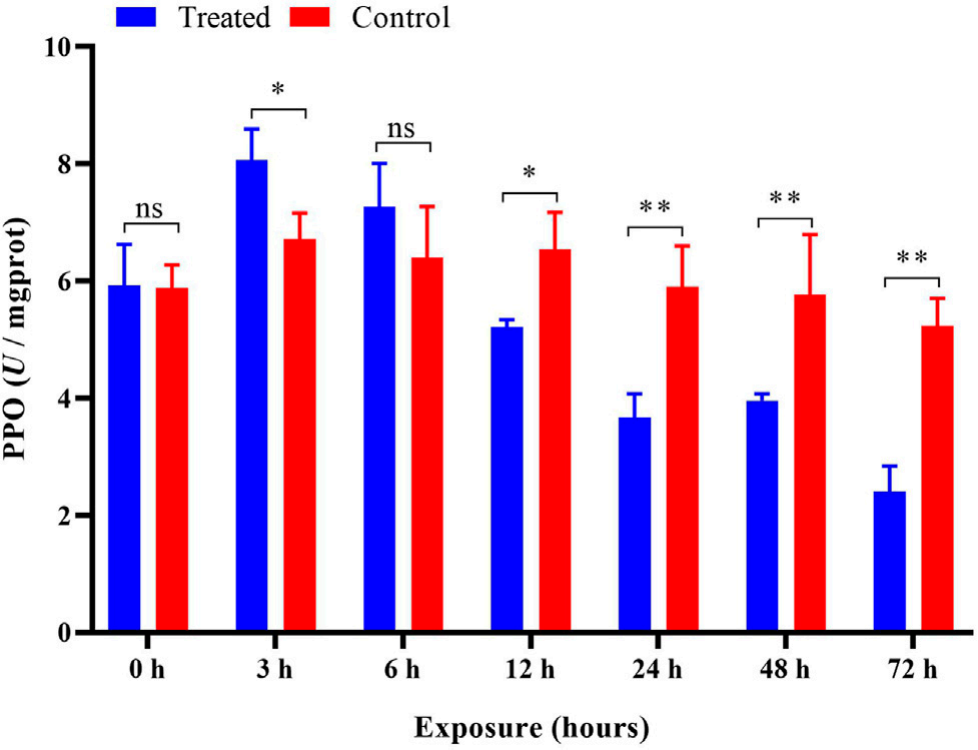
Effects of imidacloprid on the PPO activity of *P. analis*. Each bar represents the mean of three replicates, and the error bars represent the standard deviation (SD). Significant values (*p < 0.05, **p < 0.01) refer to the difference between exposed samples and the controls.

After imidacloprid exposure, the PPO activity was significantly higher (*p* < 0.05) than that of the control after 3 h of exposure. In addition, there was a significant decrease in PPO activity in the treatment groups after 12 h indicating an inhibition of the PPO activity (Figure 8). Our ANOVA revealed significant influences of different duration (*p* < 0.05) on the activity of PPO, and the trend of PPO activity variation was similar to that of POD activities in *P. analis* larvae infected with imidacloprid; however, the peak value appeared at 3 h, which was 3 h earlier than the changes of POD activities (Table 1).

## Discussion

Fireflies are an ideal environmental indicator species for pollution and insecticides due to their susceptibility to pesticides and flashing behaviors (Lewis et al., 2020; Colares et al., 2021; Pearsons et al., 2021; Zhang et al., 2021). Thus, our study investigated the physiological and histological changes in response to imidacloprid and characterized their luminant characters following the imidacloprid exposure. The imidacloprid exposure concentration used in the present study also represented the residual concentrations of imidacloprid in the environment in some regions (0.1 mg/L to over 0.32 mg/L) (Van Dijk et al., 2013; Chen M. et al., 2015; Li et al., 2018). The larvae of *P. analis* exposed to a dose of 0.1 mg/L imidacloprid showed an obvious toxic response within 6 h of exposure, however, only a few died within 72 h. Pearsons et al. (2021) also obtained a similar result after measuring the toxic response and mortality of Eastern North American fireflies when exposed to clothianidin.

In our study, persistent luminescence in the larvae of *P. analis* was observed after 12 h of exposure to imidacloprid. This phenomenon could be related to imidacloprid binding to the nAChRs, which caused the continuous conduction of nerve impulses (Topal et al., 2017; Cartereau et al., 2021). However, some studies reported that bioluminescence could be an auxiliary oxygen detoxifying mechanism in fireflies (Barros and Bechara, 1998; Dubuisson et al., 2004). Bioluminescence is a reaction that requires oxygen (Lambrechts et al., 2014), and reactive oxygen species and superoxide played crucial roles in bioluminescence (Luo and Liu, 2015). Studies have shown a link between bioluminescence and oxygen-free radical metabolism (Richter, 1977; Timmins et al., 2001; Nazari et al., 2013), moreover, antioxidant enzymes and luciferase could cooperate to minimize the oxidative stress (Barros and Bechara, 2001). Therefore, in our study, the persistent luminescence of larvae was likely caused by an excess reactive oxygen species in their body. The first significant increase in the MDA content was detected at 12 h after the imidacloprid exposure, and this coincided with the start of the continuous glow. The level of MDA at 48 h was significantly lower than that at 24 h after exposure to imidacloprid. According to Barros and Bechara (1998), the decline in the MDA content was caused by bioluminescence and antioxidant enzymes that cooperated to minimize the oxidative stress. With increasing exposure time in the treatment, gradual dimming to the extinction of luminescence from larvae was observed over 48–72 h. This could be due to damage to the biological system caused by excess ROS (Toyokuni, 1999; Juan et al., 2021).

In our study, there were significant impairment in midgut cells and fat body cells after 48 and 72 h of imidacloprid exposure. Overall, the observed histological profiles in the fat body cells and midgut tissue included the different shapes of cells and the level of cytoplasmic homogeneity. These changes may be caused by excess ROS in the organisms as organisms can produce excessive ROS under the stimulation of imidacloprid (Janner et al., 2021). The increased levels of ROS lead to oxidative stress, gastrointestinal damage (Xiao et al., 2006), lipid peroxidation, and cell damage (Davalli et al., 2018). Thus, significant oxidative damage was observed in the tissues and cells in our study while ROS in eukaryotic cells is commonly produced during cellular respiration (Juan et al., 2021. In previous studies, similar damage was also reported in honeybees (*Apis mellifera* L.) after 72 h of pesticide exposure (Pervez and Manzoor, 2020). Dittbrenner et al. (2011) showed that there was significant damage to the midgut tissue in *Eisenia fetida* for all imidacloprid exposure times (1, 7, and 14 days). Moreover, cell membranes are sensitive to radical damage due to the presence of polyunsaturated fatty acids. Lipid peroxidation occurs when ROS contacts with membrane phospholipids. Lipid peroxidation causes changes in the membrane structure, damaging the integrity of cells, and imposing oxidative stress resulting in apoptosis (Yadav et al., 2019). This result was also reflected in the morphological changes of fat body cells in this study.

The enzyme AChE catalyzes the hydrolysis of acetycholine, and it is involved in important functions such as biological nerve function regulation and muscle movement (Hemingway et al., 2004). Previous reviews have demonstrated that AChE is induced by exposure to the sublethal doses of neonicotinoids, and it can be used as a biological indicator to identify pesticide residues in terrestrial and freshwater aquatic systems (Fulton and Key, 2001; Moreira et al., 2001; Yadav et al., 2009). In the present study, the activity of AChE was significantly increased at 6 h compared with the control after imidacloprid exposure. The increase in AChE activity may be attributed to the imidacloprid binding to the insect nicotinic receptor (Li et al., 2017). However, compared with the control after imidacloprid exposure, the activity of AChE was markedly inhibited from 12 to 72 h. The inhibitory effects indicated that sublethal imidacloprid could cause harmful damage to the biochemical metabolism of *P. analis*. Li et al. (2017) also obtained a similar result after measuring the AChE activity of *Apis mellifera* and *Apis cerana* when exposed to imidacloprid at different times. During exposure to imidacloprid, AChE activity increased from 0–to 6 h and then decreased after 12–72 h in our study. This phenomenon is mainly related to the morphology of AChE in larvae and the induction of imidacloprid metabolites on target enzymes (Suchail et al., 2001; Tomalski et al., 2010). The key point of AChE active variation in the present study corresponds with the half-life of imidacloprid (5 h) in bees (Suchail et al., 2004). Therefore, the variation of AChE activity may be associated with the half-life of imidacloprid. The result was consistent with the report of Jin et al. (2015), who found that the sublethal imidacloprid could make the activity of AChE in *Apis mellifera* rise and then fall within 24 h. We feel the effect of imidacloprid on AChE in *P. analis* should be subjected to further investigation.

Previous reviews have demonstrated that pesticides induced oxidative stress leading to the generation of free radicals which caused lipid peroxidation (El-Gendy et al., 2010). The antioxidant enzymes play a vital role in defending against free radicals in organisms and they are the first line to prevent the damage of ROS to the biological system (Rodriguez et al., 2004; Hong et al., 2020). In the antioxidant system of the insects, the functions of SOD, CAT, and POD are to remove excess oxygen free radicals and maintain the redox balance (Felton and Summers, 1995; Wang et al., 2020). Some reviews have reported that slight oxidative stress can induce these antioxidant enzymes (Zhang et al., 2013; Zhang et al., 2014). However, severe oxidative stress can damage the metabolic mechanism of biological systems, causing the inhibition of these enzymes (Siddique et al., 2007; Zhang et al., 2014; Wang et al., 2016; Li et al., 2017; Jameel et al., 2019).

In this present study, we tested the activity of four antioxidant enzymes (SOD, CAT, and POD) in *P. analis* at different times after exposure to imidacloprid. SOD can remove the superoxide (O_2_^−^) to H_2_O_2_ and protect the cells from oxidant damage. The results presented have shown that the activity of SOD was significantly increased after 3–24 h of exposure to imidacloprid. This phenomenon indicated that sublethal imidacloprid could induce the biosynthesis of SOD in *P. analis* in the early stages of exposure to pesticides. This result was consistent with the report of Yucel and Kayis (2019), who found that imidacloprid could increase the SOD activity in *Galleria mellonella* L. However, with increasing exposure time, the activity of SOD was significantly decreased from the peak, and SOD activity was inhibited compared with the control after 72 h of exposure. This result indicated that overt oxidative stress had occurred and SOD fails to scavenge the overproduction of ROS. According to Wang et al. (2016), the main reason for the decline in SOD was the excess O_2_^−^ which inhibited the synthesis of SOD. Ge et al. (2015) showed that the trend of SOD activity variation was due to the metabolism of imidacloprid over time. Siddique et al. (2007) reported that the excessive ROS could render protective mechanisms of the cell ineffectively and inactivate the SOD.

SOD can catalyze the conversion of O_2_ to H_2_O_2_ further detoxified by CAT and POD. CAT is an important antioxidant enzyme, which can scavenge H_2_O_2_ to H_2_O and O_2_ (Koivula et al., 2011). In the present study, the trend of CAT activity variation was similar to the SOD activity in *P. analis* larvae infected with imidacloprid. This phenomenon could be explained by the synergistic effect of SOD and CAT (Zhang et al., 2013). With the antioxidant reaction of SOD, the content of H_2_O_2_ increased, to maintain the balance of hydroxyl radicals (-OH) in the organism, and the activity of CAT also increased accordingly (Ge et al., 2015). Moreover, compared with the control, we observed a sharp decline in SOD and CAT activities at 48 h as compared to 24 h and the CAT activity was inhibited significantly (*p* < 0.01) at 72 h in the treatments. These results could be attributed to enzyme synthesis, inactivation, or assembly of its subunit modification caused by ROS (Verma and Dubey, 2003; Batista-Silva et al., 2019; Jameel et al., 2019). Wang et al. (2016) reported that CAT could be inhibited even under the stress of low-concentration imidacloprid for a long time.

POD is the enzyme that catalyzes the oxidation of substrates with hydrogen peroxide as an electron acceptor. It mainly exists in the peroxisome of the carrier, and it has the dual effect of eliminating the toxicity of hydrogen peroxide and phenols, amines, aldehydes, and benzene (Sunde and Hoekstra, 1980). In the present study, the POD activity was increased at first from 0 to 6 h, although the difference was not significant at 3 h compared with the control, and then decreased from 6 to 72 h. This variation showed that at the beginning of imidacloprid exposure, the POD activity was activated, but the POD activity gradually decreased with the extension of the exposure time. The result was consistent with the report of Zhang et al. (2014), who found that a relatively low concentration of imidacloprid could increase the activity of POD in *Eisenia fetida* over short-term exposures. Zhang et al. (2013) also obtained a similar result after measuring the POD activity of *E. fetida* after exposure to fomesafen at different times.

MDA is the end product of lipid peroxidation, and the MDA level may also indicate the level of ROS (Chen J. et al., 2015) and the extent of cell tissue trauma (Zhang et al., 2013). In the present study, the content of MDA did not change after exposure to imidacloprid at 0, 3, and 6 h. However, it was significantly increased after 12–72 h of exposure as compared to the control. A possible reason for this observation is connected to the ROS content and antioxidant enzyme activity (Wu and Liu, 2012; Wang et al., 2016). At the beginning of exposure to imidacloprid, stimulation of pesticides increased the activity of antioxidant enzymes in organisms (Zhang et al., 2014; Li et al., 2017). However, with the extension of the exposure time, the MDA content in organisms increased gradually due to the accumulation of ROS (Balieira et al., 2018). The increase in the MDA content showed that lipid peroxidation could be caused by sublethal imidacloprid in *P. analis*. Many studies have shown that membrane lipid peroxidation is caused by ROS and this induced many negative effects (Bhattacharyya and Datta, 2001; Schaffazick et al., 2005; Balint et al., 2021). The results of the present study also confirm this point and our results also indicate that MDA has an obvious indicating effect in *P. analis* even under the stress of low concentrations of imidacloprid.

PPO is a copper-containing oxidase that oxidizes monohydric and dihydric phenols to quinones (Zhang and Sun, 2021). PPO is a crucial enzyme for melanin synthesis in insects and usually exists in insect hemolymph in the form of zymogen (Lu et al., 2014). It is activated and hydrolyzed by specific serine protease cascade to generate active PPO, which plays an important role in insect immune responses (Jiang et al., 1998; Xu et al., 2020; Ma et al., 2010). This enzyme could be an indicator for assessing the toxicity of pesticide residues (Lu et al., 2014). Magierowicz et al. (2020) reported that *Tanacetwn vulgare* essential oil significantly increased the PPO activity in larvae of *Acrobasis advenella* (Lepidoptera: Pyralidae). Ma et al. (2010) showed that cantharidin could significantly inhibit the activity of PPO in the fifth instar larvae of *Mythimna separata*. The results of the present study show that the activity of PPO increased at first and then decreased after imidacloprid exposure. This result was consistent with the report of Li et al. (2017), who found that PPO activity in *Apis mellifera* increased first and then gradually decreased with prolonged time after exposure to sublethal doses of imidacloprid. The PPO is an important immune protein in insects and mediates humoral immunity (Fan et al., 2016). In the present study, we found sublethal imidacloprid inhibited the activity of PPO significantly over 12 h. The inhibition could cause insects to be more susceptible to infection by bacteria (Zhao et al., 2007), fungi (Yassine et al., 2012; Dubovskiy et al., 2013), and some other microorganisms (Binggeli et al., 2014).

The impacts of pesticides on insect fitness have been extensively studied, and some pesticides reduced the fitness of bee larvae of both *Apis mellifera* and *Apis cerana* (He et al., 2022). Even sublethal imidacloprid can affect foraging and colony fitness in *Monomorium antarcticum* and *Linepithema humile* (Barbieri et al., 2013). In particular, neonicotinoid insecticides may reduce the fitness by impairing sperm and hypopharyngeal glands (Minnameyer et al., 2021). As imidacloprid used in our study is a member of the neonicotinoid group, it caused toxicity to firefly larvae implying that imidacloprid might reduce the fitness of firefly even at a very low dose.

Previous studies found that the fitness of the insect could be affected by the antioxidant enzyme activity (Dhivya et al., 2018), and a reduction in antioxidant enzyme activity was accompanied by a decrease in insect fitness (Slos et al., 2009; Xie et al., 2015; Cai et al., 2018). The possible reason for a reduction in fitness was impaired digestion and absorption caused by the released free radicals when the antioxidant enzyme activity was reduced (Rahimi et al., 2018). Similarly, we found that the antioxidant enzyme activity of *Pyrocoelia analis* was inhibited significantly after the treatment of low dose imidacloprid. These results implied that residues of imidacloprid in the environment affect the fitness of fireflies.

As a widely used chemical pesticide in a range of agro-ecosystem, imidaclorpid residues could be easily spread throughout the whole ecosystems. Although there were many species in the agro-ecosystem affected by the imidacloprid residues that we previously discussed, very few studies have focused on the effects of this chemical on remote regions and other ecosystems. This study characterized the toxicology of a sub-lethal dose of imidacloprid for fireflies that inhabit a forest ecosystem. The very low dose of imidacloprid caused irreversible physiological changes to the fireflies and overt toxicity implying that the chemical pesticide residues affected the whole ecosystem and not only the agroecosystem where it was applied. This potential threat to ecosystem health has not been seriously addressed by the region’s agricultural administration or by farmers.

## Data Availability

The original contributions presented in the study are included in the article/Supplementary Material; further inquiries can be directed to the corresponding author.
